# LARCalc, a tool to estimate sex- and age-specific lifetime attributable risk in populations after nuclear power plant fallout

**DOI:** 10.1038/s41598-023-46964-6

**Published:** 2023-12-01

**Authors:** Jonathan Sundström, Mats Isaksson, Christopher L. Rääf

**Affiliations:** 1 Department of Medical Radiation Sciences, Institute of Clinical Sciences, Sahlgrenska Academy, University of Gothenburg, Gothenburg, Sweden; 2 Medical Radiation Physics, Department of Translational Medicine, Lund University, Malmö, Sweden

**Keywords:** Risk factors, Environmental impact, Nuclear physics

## Abstract

A tool called LARCalc, for calculating the radiological consequences of accidental large scale nuclear power plant releases based on estimates of ^137^Cs ground deposition, is presented. LARCalc is based on a previously developed models that has been further developed and packaged into an easy-to-use decision support tool for training of decision makers. The software visualises the radiological impact of accidental nuclear power plant releases and the effects of various protective measures. It is thus intended as a rapid alternative for planning protective measures in emergency preparedness management. The tool predicts projected cumulative effective dose, projected lifetime attributable cancer risk, and residual dose for some default accidental release scenarios. Furthermore, it can predict the residual dose and avertable cumulative lifetime attributable risk (LAR) resulting from various protective measures such as evacuation and decontamination. It can also be used to predict the avertable collective dose and the increase in cancer incidence within the specified population. This study presents the theoretical models and updates to the previous models, and examples of different nuclear fallout scenarios and subsequent protective actions to illustrate the potential use of LARCalc.

## Introduction

The consequences of a nuclear power plant (NPP) accident, resulting in the release of radionuclides to the environment, will largely depend on the protective measures implemented soon after the accident. According to international recommendations, such actions should be planned in advance^1^. Comprehensive decision support systems are available today (e.g. ARGOS^2^ and JRODOS^3^), intended for the assessment of environmental and radiological consequences for various scenarios involving atmospheric dispersion of fission products from nuclear facilities. It is challenging to estimate the individual radiation doses from radioactive fallout due to the numerous factors that influence the distribution of doses. These factors include the actual NPP fallout scenario (radionuclides, activities, and variation over time), the atmospheric dispersion of the radioactive material, the areal distribution of deposition and whether fallout occurs by wet or dry deposition, the type of land use and ecosystems in the area, local and regional food production, human behaviour patterns, residence times in different environments etc.^4^.

An important component of nuclear fallout in the case of an atmospheric release in which fission products and fuel particles escape filtration systems is the fission product ^137^Cs, as the deposition density, *A*_*dep*_ (kBq m^-2^), dictates the long-term habitability in the affected area. It may be difficult to measure the actual ^137^Cs deposition following an accidental release from a NPP by uncollimated in situ gamma spectrometry, even using modern-day digital amplification to mitigate pulse pile-up in high-dose-rate environments^5^. However, it may still be feasible to determine the ground deposition at a relatively early stage after the accident, for example, by means of unmanned or conventional airborne gamma spectrometry^6^. Regardless of the radiometric techniques used, emergency preparedness strategies in most countries will be focused on the ability to estimate and report values of *A*_*dep*_ of ^137^Cs. The authors of this paper have previously developed models that aggregate external and internal exposure pathways from fallout to humans from radioactive fallout^7–10^, enabling the estimation of the long-term radiation dose from initial measurements of the ground deposition of the gamma-emitting fission product ^137^Cs.

In the previous models (see Eq. 1 and 2), the radiation dose was determined for a NPP fallout scenario representing the Chernobyl accident’s fallout in Sweden (e.g. IAEA^11^ and UNSCEAR^12^). This was done by relating the initially measured ambient equivalent dose rate from all gamma-emitting radionuclides in the fallout to the measured initial ground deposition of ^137^Cs in terms of a proportionality factor, *d*_*Cs*_^13^. The weathering process and gradual ground penetration of the radionuclides was taken into account by applying element-specific ecological half-times to obtain the predicted variation of the ambient equivalent dose rate over time. Furthermore, in the wake of the ICRP publication 144^14^, in which nuclide-specific conversion factors between ground deposition and the corresponding dose rate contribution are presented in terms of effective dose rate, air kerma and ambient dose equivalent rate, the previous models have been further developed and coded as a MATLAB app. This app, and the underpinning models, are referred to as the tool LARCalc.

This paper presents the extended features implemented in LARCalc, including the management of a variety of radionuclides associated with nuclear power generation and with nuclear detonations fallout, using the gamma emitter ^137^Cs as a key nuclide. This enables the modelling of the risk to the public for various fallout scenarios. These can be matched to a larger number of potential nuclide vectors than in the previous models, which were based on specific releases from Chernobyl and Fukushima fallout. Furthermore, we exemplify some typical risk assessments and the impact of various protective measures, and combinations thereof, in terms of the averted cumulative attributable risk and residual dose, and the way in which LARCalc can visualise the effects of these parameters for specific age and sex cohorts. Note that LARCalc mainly is intended to be used as a tool for training of decision makers. The tool can also be used to plan for protective actions before an NPP accident. LARCalc is therefore not intended to be used for real-time decision support in connection to an on-going accidental release.

## Model and scenarios

### Description of the previous basic models

**Figure 1:**
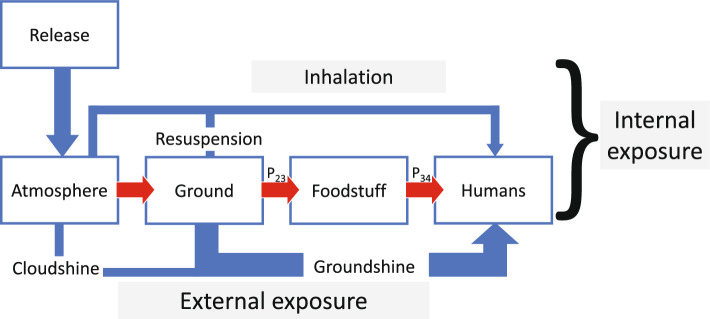
A general model for the prediction of long-term radiological consequences of atmospheric nuclear power plant fallout, including the exposure pathways external groundshine and internal committed dose by ingestion of contaminated locally produced foodstuff.

Provided the relation between the initial nuclide-specific ground deposition (kBq m^-2^) and the release of ^137^Cs has been established, the ground deposition of ^137^Cs can be used as a proxy for both the long-term external exposure of local inhabitants and the internal exposure due to potential long-term transfer of radiocaesium to the population if no protective measures were implemented^13^. Figure 1 illustrates a general model with multiple underlying sub-models for the prediction of the cumulative effective dose (*CED*) and lifetime attributable risk (*LAR*) from a given NPP fallout scenario using ^137^Cs as a key nuclide. This general model combines the results from different sub-models that are designed to estimate radiation dose and risk from specific pathways of exposure, such as groundshine (exposure of radiation from nuclides on or in soil), ingestion of contaminated foodstuffs, inhalation and cloudshine (exposure of radiation from nuclides in the plume). By integrating these sub-models, the comprehensive model provides a completer and more accurate estimate of the total radiation dose and risk received by a population or individual. This general model aggregates the long-term internal and external exposure of a population to NPP fallout, based on quantities that can be determined in the early phase of the accident. The general model thus includes empirical relationships between local and regional ground deposition and time-integrated internal doses that have previously been established under various conditions regarding climate zone and food restrictions^8,15–17^. The sub-models included in the LARCalc tool also include settings to express the exposure as age-dependent organ-absorbed doses, and can convert these into LAR, as defined by the EPA^18^. The LAR represents the estimated radiation-induced lifetime risk of suffering from cancer in a certain organ at some point in life. The models implemented in LARCalc are a further development of the one presented in detail by Rääf et al.^9^.

In the previous version of the model, two parameters were defined to describe the relationship between the local ground deposition density of ^137^Cs, *A*_*dep,Cs-137,loc*_ (kBq m^-2^), and the corresponding external dose rate from all gamma-emitting fission products, namely the initial ambient dose equivalent rate at fallout (*t* = 0), *d*_*Cs*_ (mSv y^-1^/(kBq m^-2^)), and the variation of the external dose rate over time, *r*(*t*). The latter, *r*(*t*), was observed to follow a four-component exponential decay function in terms of half-times^7^. However, *r(t)* is normalized to the initial external dose rate at *t* = 0, so it needs to be combined with *d*_*Cs*_ to describe how the external dose rate per unit initial ^137^Cs ground deposition changes over time, in other words *r(0)* = 1 (unitless).

In addition to the external exposure pathway following large-scale NPP fallout, various transfer pathways will cause contamination of locally and regionally produced foodstuff, such as dairy products, meat, vegetables, freshwater fish, mushrooms, berries, wild game, etc. In previous studies on the whole-body burden of radiocaesium in different population cohorts after nuclear weapons fallout and Chernobyl fallout in 1986, it has been shown that the average internal contamination of radiocaesium in a population can be predicted by the regional average deposition of ^137^Cs, *A*_*dep,Cs-137,reg*_ (kBq m^-2^), combined with time-integrated aggregate transfer factors, sometimes also referred to as radioecological sensitivity^17,19,20^. These transfer pathways can be represented by the convolution of transfer pathways P_23_ and P_34_ in Fig. 1. Once the average ^137^Cs body concentration has been established, transient equilibrium in the intake and excretion of radiocaesium can be assumed after one year. The committed effective dose (accounting for the biokinetic fate of radiocaesium when ingested) can then be related to the corresponding organ-absorbed dose rate and effective dose rate^21^.

In our previous model^9^, the general expression for the *CED* (mSv) from combined external and internal exposure arising from the initial ground deposition of nuclear fallout containing ^137^Cs with accompanying short-lived fission products and ^134^Cs, was given by the following expression:1where *t*_*acc*_ (y) is the time considered for integration of the dose rate after the initial fallout at *t* = 0. In Table 1 is given a further explanation of the other parameters. Note that the first term on the right-hand side of Eq. (1) refers to the external dose contribution from the local ground deposition, *A*_*dep,Cs-137,loc*_ (kBq m^-2^), and the second term refers to the internal dose contribution from radioecological transfer of regional deposition of ^137^Cs (represented by the quantity *A*_*dep,Cs-137,reg*_ (kBq m^-2^)).

**Table 1:** Parameters used in the previous models for calculation of the *CED*, and *CUMLAR* resulting from fallout from a nuclear power plant. Note that some of the parameters are still used in the updated models (see Sect. "Updates and extended features of LARCalc").

Parameter	Description (unit)
${{A}}_{{dep},{Cs}-137,{loc}}\left({x},{y}\right)$	Average local deposition of ^137^Cs (kBq m^-2^) at the dwelling coordinates (x and y), decay-corrected to the time of the fallout event. This quantity is often obtained through airborne gamma spectrometry mapping used e.g. in geological surveys
${{A}}_{{dep},{Cs}-137,{reg}}$	Average regional deposition of ^137^Cs (kBq m^-2^), decay-corrected to the time of the fallout event, can be attributed to the area over which fresh milk and meat are produced. This area was typically the county level (~ 10.000 km^2^) in Sweden at the time of the Chernobyl fallout in 1986
${{\varnothing }}_{{K}/{H}}\left(600\mathrm{keV}\right)$	Ratio between air kerma rate and ambient dose equivalent rate 1 m above ground for an infinite uniform surface deposition of gamma emitters with photon energy 600 keV (mGy mSv^-1^). A value of 0.83 was used in the previous model
*C*_*E*/*K*_	Ratio between effective dose rate and air kerma rate, in mSv mGy^−1^, at 600 keV (See also Rääf et al.^9^)
*f*_*snow*_	Snow cover shielding factor (dimensionless), averaged over the whole year for the ambient dose rate 1 m above ground. In this study, no snow cover was included, and f_snow_ was thus set to unity
*r(t)*	Time-dependent function describing the decrease in external ambient dose equivalent rate 1 m above ground, normalized to the maximum initial dose rate following NPP fallout corresponding to Chernobyl-like wet deposition at locations remote from the release point. Apart from external gamma contributions from ^134^Cs and ^137^Cs, corresponding contributions from gamma emitters such as ^131^I, ^132^I, ^132^Te, and ^140^Ba, are included^7^ A time-dependent function composed of four components was taken from Jönsson et al.^7^, with time constants expressed in terms of y^-1^ (Eq. 1)
*f*_*out*_	The fraction of time spent outdoors by an individual residing in a temperate climate zone. Typical values range between 0.1 and 0.2 for Northern European populations^25^. A value of *f*_*out*_ = 0.2 was used in this and previous work
*f*_*shield*_	Shielding factor due to indoor shelter, ranging between 0.1 and 0.4 for Northern European houses^26^. A value of *f*_*shield*_ = 0.4 was used in this work
*t*_*acc*_	Time over which the radiation exposure is integrated (y)
*t*	Time in y
*T*_*1*/*2,Cs-137*_	Physical half-life of ^137^Cs = 30.2 y
*T*_*1*/*2,Cs-134*_	Physical half-life of ^134^Cs = 2.06 y
*S*_*decont*_	Factor representing the ratio between the ambient dose equivalent rate in the area after and before a decontamination procedure. Since the calculations in this study refer to unmitigated conditions with no protective measures, *S*_*decont*_ is by definition unity
*FR*	Initial activity ratio between ^134^Cs and ^137^Cs in the fallout
${{d}}_{{i}}\left(\beta \left({t}=0,{age}\left({t}\right)\right)\right)$	Organ-specific external absorbed dose rate per unit deposition of the radionuclide in the nuclear fallout for an individual of a given age and sex at time *t* after the fallout. The age- and sex-dependent organ-specific coefficients were taken from the ICRP^14^
*k*_*Organ,int,Cs-134*_ and *k*_*Organ,int,Cs-137*_	Ratio between the organ-absorbed dose and the average whole-body absorbed dose resulting from a uniformly distributed internal contamination of ^134^Cs and ^137^Cs, respectively (explained more extensively in Rääf et al.^9^)
*T*_*ag,max,Cs*_	Amplitude factor of the aggregated transfer over all radioecological transfer pathways. This parameter determines the magnitude of the time-dependent transfer function, *T*_*ag,max,Cs*_*(t)* (Bq kg^−1^)/(kBq m^−2^), from regional-average ground deposition to whole-body concentration of ^134,137^Cs in residents. The value of *T*_*ag,max,Cs*_ thus varies from 6.7 in the general population to $\sim$ 20 (Bq kg^−1^)/(kBq m^−2^) for hunters and > 115 (Bq kg^−1^)/( kBq m^−2^) for reindeer herders in Sweden. An overview of the parameter *T*_*ag,max,Cs*_ and the time constants *t*_*1*_, *t*_*2*_, and *t*_*3*_ for different Swedish populations is given in Isaksson et al.^8^
*S*_*aliment*_	Factor representing the relative decrease in proportion to the standard radioecological transfer factor of foodstuffs resulting from various protective measures. Since the calculations in this study refer to unmitigated conditions with no protective measures, *S*_*aliment*_ is by definition unity
*f*_*sex*_	Empirical factor accounting for the lower observed radiocaesium concentration per unit body mass in females than in males (Rääf et al., 2006a). *f*_*sex*_ = 0.61 for female aged ≥ 20 y; *f*_*sex*_ = 1 for male of all ages and female < 20 y. An average sex factor of *f*_*sex*_ = 0.81 (mean value for adult men and women) was used for the estimation of *CED* in our previous studies
*e*_*Cs-137*_*(w)*	The effective dose rate conversion coefficient (mSv y^−1^/(Bq kg^−1^)) taken from Falk et al.^27^. This is expressed as *e*_*Cs-137*_*(w) = 0.0014∙w(age(t))*^*0.111*^, where the factor 0.0014 is a constant obtained from curve fitting and *w(age)* (kg) is the mean body weight of an individual of a certain age. It is assumed that this quantity is numerically equal to the absorbed dose rate per unit activity concentration in the body (see also Rääf et al.^9^)
*e*_*Cs-134*_*(w)*	Likewise for ^134^Cs, expressed as *e*_*Cs-134*_*(w) = 0.00164∙w(age(t))*^*0.188*^
*w(age(t))*	Body mass (kg) as a function of age. A fit to the data given by Wikland et al.^28^ (see also Rääf et al.^9^)
*t*_*1*_, *t*_*2*_ and *t*_*3*_	Time constants for radioecological transfer depending on type of population. Values used here are *t*_*1*_ = 1.0 y, *t*_*2*_ = 0.75 y, and *t*_*3*_ = 15 y. Values for other types of populations can be found in Isaksson et al.^8^
*c*_*1*_ and *c*_*2*_	Weighting factors for the above time constants for radioecological transfer. Values used in this study are *c*_*1*_ = 1 and *c*_*2*_ = 0.1
${{k}}_{{SEQ},{Organ},{ext}}$	Organ-specific absorbed dose rate per unit kerma rate 1 m above ground for an adult of sex female or male. Values for the organs related to cancers specified in EPA^18^ are given as sex-specific *k*_*SEQ,Organ,ext*_ (Gy Gy^−1^) and were taken from Zankl et al.^29^
${{k}}_{{SEQ},{K}}$	Age-dependent organ-specific absorbed dose rate per unit kerma rate, normalized to the corresponding value for an adult (female or male, respectively). The age-dependence curve for the thyroid (Rääf et al.^23^) was assumed to be applicable for all organs:${{k}}_{{SEQ},{K}}\left({age},{t}\right)=$ $$\left\{ {\begin{array}{*{20}l} {\left( \begin{gathered} 0.0015 \cdot age\left( {\text{t}} \right)^{5} {-}0.1214 \cdot age\left( {\text{t}} \right)^{4} \ldots \hfill \\ + 3.473 \cdot age\left( {\text{t}} \right)^{3} {-}40.28 \cdot age\left( {\text{t}} \right)^{2} \ldots \hfill \\ + 136.3 \cdot age\left( {\text{t}} \right) + 1233 \hfill \\ \end{gathered} \right)/1017} \hfill & {\mathrm{for~age~} < 20} \hfill \\ 1 \hfill & {\mathrm{for~age~} \ge 20} \hfill \\ \end{array} } \right.$$

The corresponding expression for a specific organ-absorbed dose, ${D}_{org,sex}$, as a function of ^137^Cs deposition was given by:2

The cumulative lifetime attributable risk, *CUMLAR*, of radiation-induced cancer in a specific organ resulting from the external and internal exposure pathways is given by:3where ${\dot{D}}_{org,sex}$ is the organ-specific absorbed dose rate (i.e. the time-derivate of Eq. (2)) and *LAR*_*org,sex*_ is the organ- and sex-specific risk coefficient of cancer induction per unit absorbed dose, as given by EPA^18^. The remaining parameters for the given equations are given in Table 1. The equations for the empirical transfer functions between ground deposition and whole-body concentration of ^137^Cs and ^134^Cs, *T*_*ag,max,Cs*_*(t)*, are explained in Isaksson et al.^8^. These expressions have been used to estimate representative values of *CED* and *D*_*org,sex*_ for various Swedish populations^22–24^.

**Figure 2:**
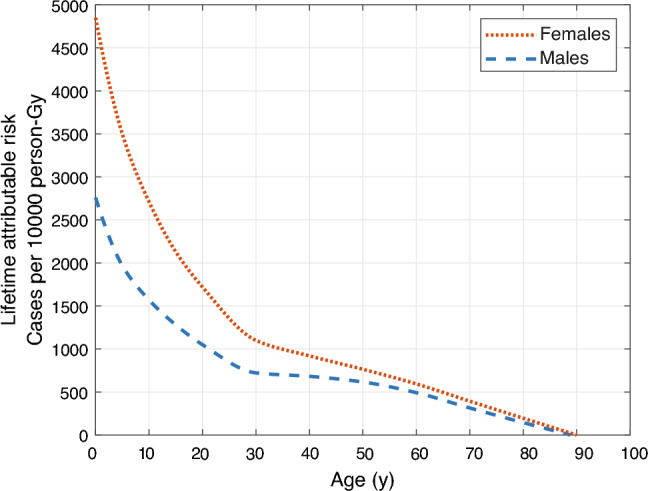
Illustration of the age- and sex-dependent lifetime attributable risk per unit absorbed organ dose for the sum of all 15 cancer types specified by the EPA, excluding non-fatal skin cancer (adopted from the EPA^18^).

The quantity *LAR*_*org,sex*_*(age)* is expressed as the probability of cancer arising from low-dose and low LET exposure, incurred at a certain age of the exposed person, during the person’s remaining life expectancy. Values of *LAR*_*org,sex*_ can be obtained from the EPA^18^ for 15 organ-specific cancers (12 for male and 14 for female) and for 5-year age classes from newborn to 80 y old. Interpolation using a piecewise cubic hermite interpolating polynomial (PCHIP)^30^ was used to obtain a continuous expression from the age- and sex-specific values tabulated by the EPA^18^ (see Fig. 2).

### Description of protective measures

When comparing the potential impact of accidental fallout from a NPP and the outcome of protective measures in terms of *CED* and *CUMLAR* for representative individuals in a population, three quantities are of special importance in order to comply with the ICRP’s recommendations^31,32^. The first is the projected radiological consequences in terms of effective dose if no protective measures are implemented (Eq. 1) and the second is the time integration of LAR (Eq. 3). When integrated between 0 and 70 y, the quantities *CED*(*t*_*acc*_ = *70 y*) and *CUMLAR*(*t*_*acc*_ = *70 y*) can thus be referred to as the projected unmitigated *CED* and *CUMLAR* , respectively. The residual dose, *CED*_*res*_, is the amount of remaining cumulative effective dose to a representative individual in a population after a given set of protective measure has been in use. Thus, *CED*_*res*_ excludes the cumulative effective dose averted by those protective measures.

The main protective measures used to reduce external dose from groundshine, are: (i) sheltering, (ii) evacuation and (iii) clean-up, or a combination of these. Regarding internal exposure, initial indoor sheltering reduces the dose resulting from inhalation of airborne radionuclides, but the most influential protective measure will be food restrictions. As well as providing the residual dose, we have included the computation of the so-called avertable cumulative lifetime attributable risk, *CUMLAR*_*av*_, in LARCalc. This quantity, which was mainly used in the previous system for radiological protection^33^, enables a direct comparison how a given protective measure relates to the averted detriment and it can be related to the efficiency of the measure. The quantities *S*_*indoor*_, *S*_*evac*,_ *S*_*aliment*_ and *S*_*decont*_ are coarse parameters describing the average dose-reducing effects of protective measures for external and/or internal exposure. All these quantities reduce the total dose rate by a factor in the range 0 to 1, (see e.g. Equations 1, 2 and 11). All the protective measures used in the model are described in the equations below (Eq. 4 to Eq. 8).

The shielding effect of staying indoors (sheltering) is described in Eq. (4) by the so-called damping factor, *f*_*u*_, and the previously mentioned factor *f*_*shield*_. The factor *f*_*u*_ describes the damping of the internal dose rate from inhalation by staying indoors, whereas *f*_*shield*_ describes both the shielding from external dose rate resulting from groundshine, as well as the external dose rate resulting from the passing plume (cloudshine). Default values of *f*_*u*_ = 0.5^34^ and *f*_*shield*_ = 0.4^26,35^ are used in LARCalc. The overall damping effect of sheltering is given by *S*_*indoor*_ (Eq. 4a) and for inhalation by *S*_*indoor,inh*_ (Eq. 4b):4a4bwhere *f*_*out*_ is the fraction of time spent outdoors, as described in Eq. (5), and can vary between 0 to 1.5

The effect of evacuation on the effective dose rate (Eq. 1) and the organ-absorbed dose rate (Eq. 2) as a function of time is given by *S*_*evac*_ (Eq. 6). During evacuation (${t}_{ret,start}\le t\le {t}_{ret,stop}$) this will be equal to 0 and otherwise 1. Evacuation affects both internal and external pathways.6

The dose-averting effect of food restrictions on internal radiation dose is described by Eq. (7). Calculated as the effectivity of restrictions, *f*_*aliment*_ may be in the interval 1 to 0, where 0 implies 100% effective restrictions, and 1 is the level of food restrictions assumed by the Swedish authorities after the Chernobyl fallout in 1986^36^. This factor only affects the pathways for the ingestion of radiocaesium and radioiodine.7

The effect of decontamination on external radiation dose is described by the factor *S*_*decont*_ given in Eq. (8) and is dependent on the effectivity of clean-up in terms of relative instantaneous dose reduction, *f*_*decont*_. A default value of *f*_*decont*_ = 0.5 is set as default in this study, reflecting an average value of clean-up efficiency, giving a value of *S*_*decont*_ = 0.5. When *f*_*decont*_ = 0.9, representing a highly efficient clean-up measure, the value of *S*_*decont*_ will be 0.1 after clean-up at *t* = *t*_*decont,done*_. Default values are based on previous experience from Japan and Eastern Europe^37^. Linear interpolation is performed between the start (*t* = *t*_*decont,start*_) and end (*t* = *t*_*decont,end*_) of decontamination.8

### Updates and extended features of LARCalc

#### Introducing nuclide vectors

The NPP fallout scenario in connection with a release to the atmosphere can involve a number of volatile radionuclides, depending on the efficiency of the consequence mitigation systems of the NPP. In addition to ^134^Cs and ^137^Cs, radionuclides such as ^131^I, ^132^I, and ^132^Te can also be released. The initial activity ratio of a fallout radionuclide in relation to that of ^137^Cs is given by the following expression (Eq. 9).9

Table 2 lists nuclide vectors representative of four nuclear fallout scenario, normalized to the initial fallout of ^137^Cs. In the updated LARCalc, nuclide vector can be adjusted to a specific fallout scenario involving ^137^Cs and accompanying radionuclides.

**Table 2:** Initial nuclide vectors for three NPP fallout events and one fictious NPP release, normalized to the initial fallout of ^137^Cs (*A*_*dep,i*_/*A*_*dep,Cs-137*_).

Nuclide	Chernobyl 1	Chernobyl 2	Fukushima	Swedish NPP
	Swedish Chernobyl fallout^38^	Chernobyl fallout in Brjansk area, Russia^11,39^	Fukushima release Northern trace^12^	Swedish NPP release^40^
^110m^Ag	0	0	0.0028	0.00101
^140^Ba	0.8118	0.72	0	0.0403
^82^Br	0	0	0	0.0133
^144^Ce	0.058	0.26	0	0
^134^Cs	0.575	0.54	1	1.47
^136^Cs	0.231	0.27	0.17	0
^137^Cs + ^137m^Ba	1	1	1	1
^131^I	8.04	11	11.5	13.1
^132^I	0	0	0	4.37
^133^I	0	0	0	3.25
^135^I	0	0	0	0.0172
^140^La**	0.818	0.84	0	0.0274
^99^Mo	0.181	0	0	0.0307
^95^Nb**	0.0403	0.064	0	0
^97^Nb	0	0	0	0
^144^Pr**	0.058	0.26	0	0
^103^Ru	0.425	1.68	0	0
^106^Ru + ^106^Rh	0.133	0.5	0	0
^125^Sb	0	0	0	0.00217
^127^Sb	0	0	0	0.0125
^89^Sr	0	4.25	0	0
^90^Sr + ^90^Y	0	0.5	0	0
^99m^Tc	0	0	0	0.0297
^131m^Te + ^129m^Te + ^129^Te	0	0	1.1	0.325
^132^Te	0	0	0	4.24
^132^Te + ^132^I*	5.19	16.6	8	0
^95^Zr	0.00719	0.065	0	0

*In several publications the initial ground deposition of the fission products ^132^Te and ^132^I is reported in terms of ^132^Te. (T_½_ = 3.2 d) assuming secular equilibrium with ^132^I (T_½_ = 0.096 d).

**Activities of ^95^Nb, ^140^La and ^144^Pr will all accumulate until reaching transient equilibrium with their mother nuclides ^95^Zr, ^140^Ba and ^144^Ce, respectively.

#### Element-specific ecological half-time, *Eco(t)*

In this updated model the ecological element-specific half-time is defined as a separate entity that can be modified by the operator for each element. The ecological half-time is a quantity that describes the aggregated effect on external dose rate 1 m above ground, resulting from various ecological and weathering processes, such as depth migration into soil^41^. In the LARCalc tool, *Eco*_*i*_(*t*) is thus defined as a damping factor that takes into account the gradual decrease in the external dose rate to air 1 m above ground, and is expressed as a bi-exponential function in the form given in Eq. (10):10

Such a biexponential ecological damping was found for caesium nuclides by Gale et al.^42^, with a short-term component (*i* = *Cs*) of *c*_*short,Cs*_ = 0.63 (which was somewhat lower than the median value of about 0.78 found from observations by Kinase et al. for urban areas^43^), and with *T*_*eco,Cs,short*_ = 0.6 y. Jönsson et al.^7^ performed polynomial regression, *r(t)*, to estimate the contribution of radiocaesium to the total ambient dose rate equivalent (H*(10)) in the areas of Sweden that were most affected by the Chernobyl fallout after 1986. The *r(t)* was normalised to the H*(10) measured on the first day of the plume's arrival. They found a long-term component with an effective half-time of 5.5 y, corresponding to *T*_*eco,Cs,long*_ = 6.7 y. No significant short-term component for the radiocaesium contribution could be extracted from the reported data, and Jönsson et al. therefore suggested that a value of *c*_*short,Cs*_ = 0 provides a better fit to the environmental conditions in those parts of Sweden (for a detailed list of locations, see Jönsson et al.^7^).

Based on the second component in the equation for *r(t)* given by Jönsson et al.^7^, it can be deduced that the decrease with an effective half-time of 6.8 d is mainly attributed to ^131^I, which would roughly correspond to an ecological half-time of 0.125 y. Due to the relatively short half-life of ^131^I it is suggested a short-term component of *c*_*short,I*_ = 1 combined with *T*_*eco,I,short*_ = 0.125 y will adequately describe the ecological damping of the short-lived radioiodine nuclides.

Bremsstrahlung generation in soil and air from radiostrontium (including daughter nuclides) will contribute to the external dose, the effective dose rate for ^90^Sr/^90^Y being as high as 0.068 (mSv y^-1^)/(kBq m^-2^) for deposition on a plane surface^14^. However, this will decline rapidly as radiostrontium penetrates the surface and lower soil layers (< 0.001 (mSv y^−1^)/(kBq m^-2^) at a penetration mass depth of 0.5 g cm^-1^). According to Sahoo et al.^44^, radiostrontium typically migrates 1 cm y^-1^, and we therefore suggest values of *T*_*eco,Sr,short*_ of 0.15 y, and *c*_*short,Sr*_ = 1 for dry deposition, leading to an almost zero contribution to the external dose after reaching a mass depth of 0.5 g cm^−1^, assuming a soil density of 1.5 g cm^-3^. Due to the lack of comprehensive data on ecological half-times in terms of external dose rate from ground deposition for radionuclides of other elements than Cs, Sr and I, we suggest default ecological half-times of *T*_*eco,i,short*_ = 0.6 y (with a *c*_*short,I*_ = 0.63) and *T*_*eco,i,long*_ = 22.7 y, for all other radionuclides, which are the same as the values for Cs reported by Gale et al.^42^. Users of the LARCalc tool can however use specific values for the long-term and short-term ecological half-times of the various elements represented by the NPP fallout scenario radionuclide vector based on the data available. The suggested default values in the absence of any local or region-specific values are 6.7 y for radiocaesium, with options of using e.g. 3.2 y (representing the lower extreme value reported from Japan^45^), or 15 y, representing values observed in Russia^46^. Table 3 lists suggested values of the parameters *T*_*eco,i,short*_, *T*_*eco,i,long*_ and *c*_*short,i*_.

**Table 3:** Element-specific ecological half-times parameters of the contribution to the external dose rate 1 m above ground from fallout in terms of a double exponential decay function (Eq. 9) for the elements represented in the nuclide vectors in Table 2. N/A = Not applicable.

Nuclide	*c*_*short,i*_	*T*_*eco,i,short*_* (y)*	*T*_*eco,i,long*_* (y)*
^110m^Ag	0.63	0.6	22.7
^140^Ba	0.63	0.6	22.7
^82^Br	0.63	0.6	22.7
^144^Ce	0.63	0.6	22.7
^134^Cs	0	0.6	3.2 or 6.7 or 15
^136^Cs	0	0.6	3.2 or 6.7 or 15
^137^Cs + ^137m^Ba	0	0.6	3.2 or 6.7 or 15
^131^I	1	0.125*	22.7
^132^I	1	0.125	22.7
^133^I	1	0.125	22.7
^135^I	1	0.125	22.7
^140^La*	0.63	0.6	22.7
^99^Mo	0.63	0.6	22.7
^95^Nb*	0.63	0.6	22.7
^97^Nb	0.63	0.6	22.7
^144^Pr*	0.63	0.6	22.7
^103^Ru	0.63	0.6	22.7
^106^Ru + ^106^Rh	0.63	0.6	22.7
^125^Sb	0.63	0.6	22.7
^127^Sb	0.63	0.6	22.7
^89^Sr**	N/A	N/A	N/A
^90^Sr + ^90^Y**	N/A	N/A	N/A
^99m^Tc	0.63	0.6	22.7
^131m^Te + ^129m^Te + ^129^Te	0.63	0.6	22.7
^132^Te	0.63	0.6	22.7
^132^Te + ^132^I	0.63	0.6	22.7
^95^Zr	0.63	0.6	22.7

*Based on the initial slope of ambient equivalent dose rate data from Jönsson et al.^7^.

**For the pure beta-emitters ^89^Sr and ^90^Sr/^90^Y, the bremsstrahlung generates an external dose contribution that will be ecologically damped more rapidly than for gamma emitters. According to Kumar Sahoo et al.^44^ radiostrontium migrates at a rate of ~1 cm y^−1^ which, in combination with data from the ICRP^14^ suggests that the bremsstrahlung component decreases rapidly (T_eco,Sr,short_ < 1 y).

#### Inclusion of the ICRP nuclide-and organ-specific dose coefficients

In this study, the model used in LARCalc has been adapted to include the recently published nuclide-specific dose coefficients for a given fission product, relating the ground deposition, *A*_*dep*_, to the corresponding dose rate (effective, organ, air kerma and ambient dose equivalent rate 1 m above ground) by the ICRP^14^, to enable a broader range of hypothetical NPP release scenarios. The initial external dose rate during the first few days following a single fallout event will depend on the initial ground penetration depth of the nuclide $i$ in the fallout. Hence, the user must define the initial penetration (or relaxation) mass depth, $\beta$ (g cm^-2^) of the fallout. Dry deposition is associated with a shallower depth distribution in soil, whereas the wet deposition of NPP nuclides may lead to initial ground penetration of several cm^26^. In the LARCalc model, the user can choose between dry or wet initial deposition by setting $\beta$ equal to 0.0 or 1.0 g cm^-2^. If dry deposition is selected, an initial penetration depth, $\beta$ = 0.0 g cm^-2^ will be the default value assigned to all nuclides included in the nuclide vector. If wet deposition is chosen, the default ground penetration depth is set to $\beta$ = 1.0 g cm^-2^, at which LARCalc then retrieves the corresponding dose coefficient from the ICRP tabulated data^14^. This penetration depth can be considered representative of wet deposition following the Chernobyl fallout in Sweden^26^.

Petoussi-Henss et al.^48^ and the ICRP^14^ have presented age-dependent organ dose rate and effective dose rate coefficients for the uniform surface deposition of a gamma emitter for the estimation of external dose rate in environmental irradiation geometries such as a radioactive fallout. Rääf et al.^9^ used polynomials of organ dose as a function of age as correction factors to account for the higher organ dose rate to children than in adults for a specific surface deposition. The ICRP^14^ now provides age-dependent coefficients of effective dose (*e*_*i*_) and organ-specific equivalent dose (*d*_*i*_), which have been incorporated into the groundshine model. A library consisting of the dose conversion coefficients for newborns, 1-year-, 5-year-, 10-year-, 15-year-olds and adults (≥ 20 y) is defined in LARCalc, and the dose conversion factors for an individual of a specific age, *e*_*i*_*(age)* and* d*_*i*_*(age)* are then obtained by PCHIP of the tabulated data in the library. The updated external organ dose rate equation (Eq. 2) is thus as shown in Eq. (11).11

#### Updated internal organ-absorbed dose rate coefficients

The age dependence of the internal dose contribution from long-term intake of radiocaesium has been accounted for by adopting the same mathematical relationship between average body dose and body weight for a homogeneous distribution of radiocaesium body burden, as reported by Leggett et al.^49^, and later adopted by Falk et al.^27^. Isaksson et al.^21^ have recently published computed sex- and nuclide-specific organ dose coefficients for radiocaesium isotopes distributed in the human body when exposed to protracted intakes of radiocaesium. Organ-specific absorbed dose rate coefficients *δ*_*org,Cs-137,sex*_ and *δ*_*org,Cs-134,sex*_ were taken from Table 4 in Isaksson et al.^21^, but are here expressed in units of mGy y^-1^ instead. These parameters replace *e*_*Cs*_ and *k*_*Organ,int,Cs*_ in the previous model (see Eq. 2). The dose rate coefficients have thus been updated based on models described by the ICRP^30^. The calculated effective dose rate coefficients are given in Table 4.

**Table 4:** Effective dose rate coefficients calculated from sex- and nuclide-specific organ dose coefficients for radiocaesium isotopes from Isaksson et al.^21^.

	Adult male (mSv y^−1^) / (Bq kg^−1^)		Adult female (mSv y^−1^) / (Bq kg^−1^)		Sex average (mSv y^−1^) / (Bq kg^−1^)	
	^134^Cs	^137^Cs	^134^Cs	^137^Cs	^134^Cs	^137^Cs
$\upvarepsilon$*(t)*	4.14E-05	2.40E-05	5.33E-05	3.02E-05	4.73E-05	2.71E-05

The coefficients *δ*_*org,Cs-137,sex*_ and *δ*_*org,Cs-134,sex*_ are implemented in LARCalc in the aggregate transfer functions between ground deposition and internal organ-specific absorbed dose resulting from the intake of contaminated foodstuff (corresponding to the transfer parameter *P*_*25*_ defined by UNSCAR^15^) according to Eqs. (12) and (13). Note that the time integral of *P*_*25*_ is given by the product of ${\int }_{0}^{{t}_{acc}}{P}_{24} \cdot {P}_{45}\cdot dt$, and that *P*_*24*_ corresponds likewise to *P*_*23*_ and* P*_*34*_ in Fig. 1.1213

The explanations of remaining parameters are given in Table 1.

Although radiocaesium is the dominant contributor to long-term internal dose from accidental NPP fallout, initial transfer of radioiodine to humans via contaminated fresh milk can be substantial if no iodine prophylaxis is distributed to local residents^11^, or if dairy cows continue to graze (as happened in Belarus after the Chernobyl fallout in 1986). Furthermore, if fallout from nuclear weapons or fallout in areas close to a damaged NPP is considered, radiostrontium may be present in high concentrations and contribute significantly to the internal dose many decades after the fallout, as was the case after the global fallout from nuclear weapons testing in the 1950s and 60s^15^. In contrast to radiocaesium, both radioiodine and radiostrontium exhibit high organ-specific uptake in humans; iodine being highly accumulated in the thyroid and strontium, a calcium analogue, being mainly retained in bone tissue^15^.

The yearly fallout and measured ^90^Sr activities in human bone in Denmark^51^ were used to numerically obtain time-dependent aggregated transfer factors for radiostrontium. For a detailed description of the derivation of the transfer functions for radiostrontium, the reader is referred to Sundström^10^.14

*S*_*Build-up*_(*t*) is a linear function that takes into account the build-up of radiostrontium in the body in the first 3 months from the start of the fallout. *T*_*ag,Sr*_ is the age-dependent aggregated transfer factor between the ground deposition and ^90^Sr activity in human bone, normalized to the mass of calcium ((Bq g(Ca)^-1^)/(kBq m^-2^)). *c*_*Sr,short*_ and *c*_*Sr,long*_ are the short- and long-term weighting factors for the transfer parameter. *T*_*eco,Sr,short*_ and *T*_*eco,Sr,long*_ are the age-dependent ecological half-times in years for short- and long-term transfer, respectively. Age-specific values for the parameters of aggregated transfer factors and ecological half-time for strontium are given in Table 5. The coefficient *δ*_*MIRD,org,Sr-90*+*Y-90*_ (mGy y^-1^) is the sum of the absorbed organ-specific dose rates per unit activity in bone for ^90^Sr and ^90^Y, taken from MIRD pamphlet no. 11^52^. *m*_*Ca,sex*_*(age(t))* is a PCHIP fit to the age- and sex-dependent whole-body mass of calcium in kg^53^. A more comprehensive description of the derivations of the parameters in Eq. (14) can be found in Sundström^10^. Note that no protective measures have yet been included in Eq. (14). This work is still in progress, and is intended to be published separately.

**Table 5:** Age-specific parameters for the bi-exponential aggregated transfer factor and ecological half-time given in Eq. (16). Values are taken from Sundström^10^.

Parameter	Age cohort				
	> 1 month	1 month–4 years	4–19 y	19–29 y	< 29 y
*T*_*ag,Sr*_	0.2016	0.2993	0.2594	0.2465	0.2358
*c*_*short,Sr*_	1	1	1	1	1
*c*_*long,Sr*_	0.1426	0.1271	0.2112	0.2003	0.1664
*T*_*eco,Sr,short*_	0.6312	0.9663	0.3861	0.2146	0.1598
*T*_*eco,Sr,long*_	9.9998	9.4492	6.4569	6.6598	8.6654

In scenarios with NPP releases of radiostrontium, the more short-lived strontium isotope ^89^Sr may also accompany ^90^Sr/^90^Y. To be consistent with the approach used in the model for LARCalc, a similar absorbed dose rate coefficient, *δ*_*MIRD,org,Sr-89*_, to that used for the sum of ^90^Sr and ^90^Y must be defined for ^89^Sr. Since this nuclide is not listed by Snyder et al.^52^, the absorbed dose coefficient *δ*_*MIRD,org,Sr-89*_ was interpolated between *δ*_*MIRD,org,Sr-90*_ and *δ*_*MIRD,org,Y-90*_ with regard to the maximum emitted beta energy, *E*_*β,max*_. Coefficients for the effective dose rate (*ε*_*MIRD*_) were calculated for radiostrontium, following the same procedure as for radiocaesium (Eqs. 12, 13), excluding all organs except the skeleton and bone marrow. The values of *δ*_*MIRD,org,Sr*_ for the skeleton and red bone marrow, and *ε*_*MIRD*_ are listed in Table 6. Corresponding coefficients for the other organs are assumed to be negligible compared with the skeleton and red bone marrow and are therefore excluded.

**Table 6:** Organ-specific absorbed dose rate coefficients, *δ*_*MIRD,org,Sr*_, for an adult subjected to chronic intake of radiostrontium-contaminated foodstuff. Values are taken and/or adopted from Snyder et al. ^52^.

Organ defined in LARCalc	Absorbed dose rate coefficient	
	*δ*_*MIRD,org,Sr-90+Y-90*_ (mGy y^−1^)	*δ*_*MIRD,org,Sr-89*_ (mGy y^−1^)
Bone/Skeleton	0.001052	0.000536
Red bone marrow	0.000795	0.000182
	Effective dose rate coefficient	
	*ε*_*MIRD,Sr-90+Y-90*_ (mSv y^-1^)	*ε*_*MIRD,Sr-89*_ (mSv y^-1^)
Whole body	8.17E-05	2.72E-05

#### Additional exposure pathways: Internal dose from inhalation during plume passage and external dose from cloudshine

Although the model behind LARCalc was originally intended for long-term predictions of unmitigated and remediated exposures from NPP fallout, the need for coarse estimates of the magnitude of inhalation doses during plume passage, related to a certain ground deposition was identified. An additional algorithm has therefore been added so that LARCalc can provide a conservative estimate of the inhalation dose, in which the average near-ground concentration in the passing plume, *C*_*i,ave*_ (Bq m^-3^), is related to the local ground deposition of a given radionuclide, *A*_*dep,i,loc*_ (Bq m^-2^) by the equation:15where *v*_*d*_ (m s^−1^) is the dry deposition velocity^54^. For wet deposition the velocity is assumed to be infinite (due to washout) and thus giving a *C*_*i,ave*_*(t)* = 0 Bq m^−3^. This gives rise to two extremes: a conservative plume in the case of dry deposition, and an attenuated plume in the case of wet deposition. Assuming an age-dependent inhalation rate, INH(age) (m^3^ s^−1^)^55^, and using nuclide-, age- and organ specific-dose coefficients taken from the ICRP^56^, the conservative estimate of the time-integrated dose rate from inhalation, *Ḋ*_*inh*_ (mSv) is given by Eq. (16).16

The dose coefficients, *d*_*inh,i*_, relating unit intake with the committed equivalent or effective dose (Sv Bq^−1^), depend on aerosol or particle size, and whether the radionuclide is inhaled as a gas, vapour or organic molecule. This is important for iodine, as the inhalation of gaseous iodine (I_2_(g) or organic iodine, CH_3_I) results in more than double the organ dose to the thyroid compared to the inhalation of iodine bound to aerosols^56^. A detailed description of the way in which the dose contribution from inhalation of airborne radionuclides from the release plume is estimated in relation to the remaining ground deposition is given by Sundström^10^. The inhalation absorbed dose rate will hence be nuclide-, age- and organ-specific as well as being specific in terms of aerosol size in the form of activity median aerodynamic diameter (µm), AMAD, and absorption type fast (F), moderate (M) or slow (S). For a more comprehensive description see the ICRP^56^.

The algorithm for cloudshine dose uses the same average time-integrated near-ground air concentration (Eq. 15) in combination with the air submersion nuclide-, sex-, age- and organ-specific dose coefficient, *d*_*cloud,i*_*,* from the ICRP^14^. The dose rate from cloudshine, $\dot{D}$_*cloud,org*_ (mGy y^-1^) is given by Eq. (17).17

#### Additional exposure pathways: Internal dose from ingested iodine-131 from consumed milk

A rapid transfer of radioiodine through cow’s milk was observed in many places after the Chernobyl NPP accident, both in the area surrounding the damaged plant^57^, and also further away from Chernobyl^58^, resulting in high doses to the thyroid in the population. An algorithm to estimate the absorbed dose rate (mGy/y) due to the consumption of milk containing ^131^I is given in Eq. (18).18

where *TF*_*milk,grass*_ is the transfer factor from grass to milk (in this case 0.274 Bq L^−1^/Bq m^−2^),* f*_*inter*_ is the interception factor (0.1), *k*_*delay*_ is a factor correcting for the decay between milking and consumption (in this case 3.5 days, giving a factor of 0.739), *T*_*eff,grass*_ is the effective half-time of ^131^I in the pasture, in this study assumed to be 3.5 days as reported by Håkansson et al.^58^, *a(age(t))* is the age-dependent dairy milk consumption rate. The latter was determined using PCHIP of age-dependent annual intake data of 50 kg y^−1^ for 1-year-olds, 100 kg y^−1^ for 12-years-ols and 150 kg y^−1^ for 20-year-old and older (values taken from Rääf et al.^23^ and references therein). *d*_*ing*_ is the age- and organ-dependent dose coefficient for ingested ^131^I, taken from the ICRP^59^. For a more detailed description of the model, see Sundström^10^.

#### Final expression of *CED* and *CUMLAR* for updated LARCalc

The final expressions for the *CED* and the *CUMLAR* as a function of local and regional average deposition density, *A*_*dep,Cs-137,loc*_ and *A*_*dep,Cs-137,reg*_, respectively, for the combined internal and external pathways considered in the current version of LARCalc are given in Eqs. 19 and 20:1920

Thus, for a given NPP fallout scenario including the assumed local or regional average ground deposition of ^137^Cs, LARCalc predicts the anticipated unmitigated dose accumulated over a specified time period (e.g. 70 years). The corresponding residual dose, *CED*_*res*_, and averted cumulative lifetime attributable risk, *CUMLAR*_*av*_, can be found for a given set of protective measures using the S-factors for the different protective measures, *S*_*evac*_, *S*_*shield*_, *S*_*aliment*_ and *S*_*decont*_. The *CED*_*res*_ can then be compared with reference levels suggested by the ICRP for existing radiation exposures^30^, e.g. 20 mSv annual effective dose for returning evacuees, or 100 mSv residual dose for returning evacuees.

For planning purposes, the emergency preparedness coordinator of the NPP, as well as the relevant national authorities, may need to address the following questions:

- For a certain average local deposition of ^137^Cs, accompanied by other fission products resulting from a certain fallout scenario, for how long must the residents in the affected area be evacuated to ensure that the residual dose of 100 mSv or returning dose rate of 20 mSv y^-1^ is not exceeded?
- At which ground deposition, *A*_*dep,Cs-137*_, should restrictions on foodstuffs be implemented, for how long time and to which extent?
- What is the averted risk of developing radiation-induced cancer for different age and sex cohorts, especially on more sensitive sub-cohorts, in terms of *CUMLAR*_*av*_?
- Given that decontamination of evacuated zones can be important when encouraging evacuees to return to an evacuated area, what additional gain in terms of *CUMLAR*_*av*_, can be achieved by decontamination measures? How does this gain depend on the time frame and dose reduction efficiency for different age and sex cohorts?

Table 7 presents an overview of the mathematical expressions for *CED*_*res*_ (mSv), and *CUMLAR*_*av*_ (%), for some typical combinations of protective measures to decrease radiation exposure after NPP fallout. If the individuals are evacuated before the plume reaches their habitat, *S*_*decont*_ = 0 for *t* < *t*_*evac*_, where *t*_*evac*_ is the time of resettlement in the area. For *t* > *t*_*evac*_, the value of *S*_*decont*_ is selected by the operator, between 0 to 1, and represents the average external dose rate reduction resulting from decontamination measures implemented in the residential area.

**Table 7:** Examples of protective measures listed by extent and time duration and the quantities describing the outcome of protective measures, *CED*_*res*_ and *CUMLAR*_*av*_, based on sheltering, evacuation, decontamination, and extensive food restrictions.

Protective action scenarios		*CUMLAR*_*av*_	*CED*_*res*_
(1)	Indoor sheltering (*t*_*shelt*_)	*CUMLAR(t* = 0 to 70 y; *S*_*decont*_*(t)* = 1)—*CUMLAR(t = 0* to *t*_*shelt*_; *S*_*decont*_*(t)* = *f*_*shield*_-*f*_*inh*_⋅*D*_*inh*_⋅*LAR(age(t)))*	*CED*_*res*_ *= CED(t*_*shelt*_ to 70 y, *S*_*decont*_*(t)* = 1; *S*_*aliment*_ = 1*)*
(2)	Evacuation with resettlement time (*t*_*ret*_). Relaxed food restrictions as in Sweden after the Chernobyl fallout: *S*_*aliment*_ = 1. Sheltering 3 days	*CUMLAR(t *= 0 to 70 y; *S*_*decont*_*(t)* = 1*)*—*CUMLAR(t = t*_*ret*_ to 70 y; *S*_*decont*_*(t) =* 1*)*	*CED*_*res*_ *= CED(*1 to 70 y; *S*_*decont*_*(t) *= *S*_*aliment*_ = 1*)*
(3)	Evacuation until time *t*_*ret*_ = 1 y and decontamination with 50% efficiency. *S*_*aliment*_ = 1. Sheltering 3 days	*CUMLAR(t = *0 to 70 y; *S*_*decont*_*(t) *= 1)—*CUMLAR(t =* 1 to 70 y; *S*_*decont*_*(t)* = 0.5*)*	*CED*_*res*_ *= CED(t* = 1 to 70 y; *S*_*decont*_ *(t)* = 0.5; *S*_*aliment*_ = 1*)*
(4)	Evacuation *t*_*ret*_ and decontamination with 90% efficiency. *S*_*aliment*_ = 1. Sheltering 3 days	*CUMLAR(t* = 0 to 70 y; *S*_*decont*_ *(t)* = 1*)—CUMLAR(t = t*_*ret*_ to 70 y; *S*_*decont*_ *(t)* = 0.1*)*	*CED*_*res*_ *= CED(t*_*ret*_ to 70 y; *S*_*decont*_*(t)* = 0.1*)*
(5)	Evacuation *t*_*ret*_ and decontamination with 50% efficiency, with lifting of extensive food restrictions, *S*_*aliment*_ = 0, at *t*_*food*_. Sheltering 3 days	*CUMLAR(t* = 0 to 70 y; *S*_*decont*_*(t)* = 1; *S*_*aliment*_ = 1*) –(CUMLAR(t = t*_*ret*_ to *t*_*food*_ y; *S*_*decont*_*(t) *= 0.5; *S*_*aliment*_ = 0*) + CUMLAR(t* = *t*_*food*_ to 70 y; *S*_*decont*_*(t)* = 0.5; *S*_*aliment*_ = 1*))*	*CED(t = t*_*ret*_ to *t*_*food*_ y; *S*_*decont*_*(t)* = 0.5; *S*_*aliment*_ = 0*) + (CED(t* = *t*_*food*_ to 70 y; *S*_*decont*_ *(t)* = 0.5; *S*_*aliment*_ = 1*)*

## Results and examples

To illustrate the use of LARCalc, we here present some different examples of how the tool can be applied. The general set-up for the calculations of the *CED* and *CUMLAR* in these examples were mainly based on a fictious Swedish NPP fallout scenario (see Table 2).

**Figure 3:**
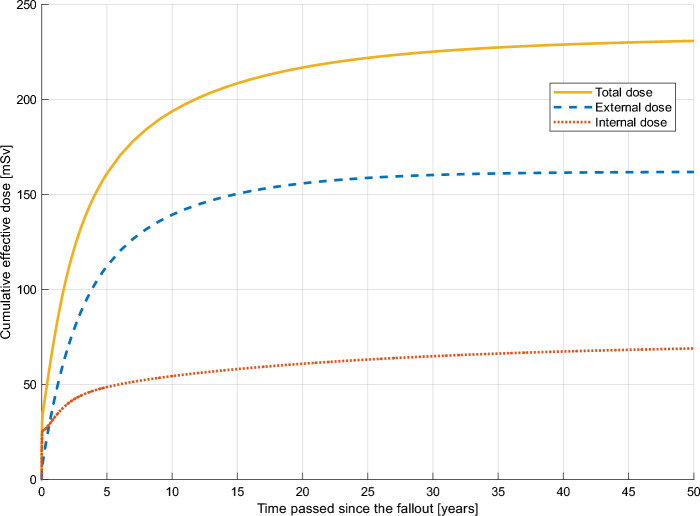
*CED* for an adult male (30-years-old at accident) after exposure fictious Swedish NPP fallout scenario (Swedish NPP in Table 2), assuming dry deposition (0 g cm^-2^). Calculated in LARCalc.

**Figure 4:**
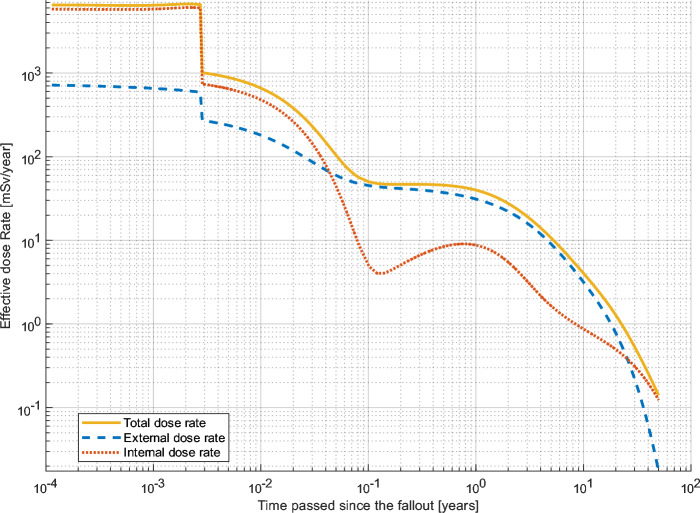
Effective dose rate for an adult male (30-years-old at accident) after exposure fictious Swedish NPP fallout scenario (Swedish NPP in Table 2), assuming dry deposition (0 g cm^-2^)as calculated by LARCalc. Note that both x- and y-axis are in log-scale.

Default values for all following accident fallout scenarios and subsequent protective actions were: no protective measures, *t*_*acc*_ = 80 y (life expectancy), *A*_*dep,loc*_ = *A*_*dep,reg*_ = 1 MBq m^-2^ of ^137^Cs, *f*_*sheild*_ = 0.4, *f*_*out*_ = 0.2, *f*_*snow*_ = 1, *T*_*eco,Cs,long*_ = 6.7 y, *T*_*ag,max,Cs*_ = 6.7 Bq kg^-1^/(kBq m^-2^), inhalation absorption type “medium” with 24 h plume passage, including all pathways*.* Any deviations from these values will be mentioned in the title of the table or figure in the following sections. Figures 3 and 4 are based on a ^137^Cs ground deposition of 1000 kBq m^-2^ both locally and regionally, assuming dry deposition, for an adult male aged 30 at the time of fallout, to an age of 80 y. In this case, no protective measures were included, corresponding to the first example in Sect. 3.1 below.

### Results of *CED*_*res*_ and *CUMLAR*_*av*_ for the different NPP fallout scenarios and protective action scenarios

#### *CED*_*res*_* and CUMLAR*_*av*_* for residents in protective action scenario (1) in *Table 7

Table 8 gives the results obtained from LARCalc and the underpinning model when computing the *CED*_*res*_ as a function of sheltering time, *t*_*shelt*_, for three different NPP fallout scenarios (Chernobyl 1, Fukushima and Swedish NPP, specified in Table 2), for dry and wet deposition. The calculations are based on Scenario (1) in Table 7. For 3-days´ sheltering, the typical residual doses will range from 106 to 230 mSv per MBq m^-2^, depending on the fallout scenario and whether the deposition of the fallout is dry or wet.

**Table 8:** *CED*_*res*_ (mSv) to a 30-year-old male for three NPP fallout scenarios (listed in Table 2) using protective action scenario (1) in Table 7. Values are presented for dry- (0 g cm^−2^) and wet deposition (1 g cm^-2^), and for various sheltering times. Note *t*_*shelt*_ = 0 d gives the effects of no sheltering.

*t*_*shelt*_	NPP fallout scenarios					
	Chernobyl 1		Fukushima		Swedish NPP	
	Dry dep	Wet dep	Dry dep	Wet dep	Dry dep	Wet dep
0 d	167.4	106.7	200.6	128.2	230.8	148.6
1 d	165.1	106.6	197.7	128.0	227.9	148.5
3 d	164.8	106.4	197.3	127.8	227.6	148.3
7 d	164.4	106.2	196.8	127.5	227.2	148.1

The residual cumulative lifetime attributable risk, *CUMLAR*_*res*_, and the *CUMLAR*_*av*_ for a newborn female and a 30-year-old male, and the corresponding *CED*_*res*_ for dry deposition of radionuclides for the three NPP fallout scenarios are given in Table 9. The *CUMLAR*_*av*_ due to sheltering will depend heavily on age, and the averted risk for a newborn female is ten times higher than for adult male. Note that *CUMLAR*_*av*_ and *CUMLAR*_*res*_ both are given in the unit percental points, or total risk of developing cancer.

**Table 9:** *CUMLAR*_*av*_ (%), *CUMLAR*_*res*_ (%) and *CED*_*res*_ (mSv) for a newborn female and a 30-year-old male for three NPP fallout scenarios (Table 2) using protective action scenario (1) in Table 7. Values are given for dry deposition, and for various sheltering times.

*t*_*shelt*_		NPP fallout scenarios					
		Chernobyl 1		Fukushima		Swedish NPP	
		Newborn female	30-year-old male	Newborn female	30-year-old male	Newborn female	30-year-old male
1 d	*CUMLAR*_*av,indoor*_ (%)	0.20	0.015	0.26	0.018	0.25	0.016
	*CUMLAR*_*res*_ (%)	21.7	2.77	25.4	3.19	29.2	3.63
	*CED*_*res*_ (mSv)	205.1	165.1	246.6	197.7	284.5	227.9
3 d	*CUMLAR*_*av,indoor*_ (%)	0.28	0.019	0.36	0.024	0.33	0.021
	*CUMLAR*_*res*_ (%)	21.6	2.76	25.3	3.19	29.1	3.62
	*CED*_*res*_ (mSv)	204.6	164.8	245.9	197.3	284.0	227.6
7 d	*CUMLAR*_*av,indoor*_ (%)	0.37	0.025	0.49	0.032	0.43	0.027
	*CUMLAR*_*res*_ (%)	21.5	2.76	25.2	3.18	29.0	3.62
	*CED*_*res*_ (mSv)	204.0	164.4	245.1	196.8	283.4	227.2

#### *CED*_*res*_* and CUMLAR*_*av*_* for residents in protective action scenario (2) in *Table 7

*CED*_*res*_ after sheltering and evacuation for one year before resettlement for three different NPP fallout scenarios, for three different ecological half-times of radiocaesium, *T*_*eco,Cs,long*_*,* assuming dry deposition, is given in Table 10. The calculations are based on the mitigated accident Scenario (2) in Table 7. *CUMLAR*_*av*_ is given for various resettlement times, *t*_*ret*_, in Table 11. It can be seen that a one-year evacuation will give the highest *CED*_*res*_ for nuclide vectors with high ^134^Cs/^137^Cs-ratios, like Swedish NPP (Table 2).

**Table 10:** *CED*_*res*_ (mSv) for a 30-year-old male for three NPP fallout scenarios using protective action scenario (2) in Table 7. Values are presented for different values of *T*_*eco,Cs,long*_*(y)* and for different evacuation times, 3 days’ sheltering, assuming dry deposition.

*t*_*ret*_	NPP fallout scenarios								
	Chernobyl 1			Fukushima			Swedish NPP		
	*T*_*eco,Cs,long*_								
	3.2 y	6.7 y	15 y	3.2 y	6.7 y	15 y	3.2 y	6.7 y	15 y
1 y	89.5	130.9	194.3	106.0	151.8	218.4	121.0	171.5	241.6
5 y	46.2	76.5	132.5	51.3	83.0	140.4	53.2	86.4	145.3
10 y	31.2	49.3	94.0	34.9	53.2	98.1	35.2	53.7	99.0

**Table 11:** *CUMLAR*_*av*_ (%), *CUMLAR*_*res*_ (%) and *CED*_*res*_ (mSv) for a newborn female and a 30-year-old male for three NPP fallout scenarios (in Table 2) using protective action scenario (2) in Table 7. Values are given for *T*_*eco,Cs,long*_ = 6.7 y, dry deposition, 3 days’ sheltering, and for different evacuation times, *t*_*ret*_.

*t*_*ret*_		NPP fallout scenarios					
		Chernobyl 1		Fukushima		Swedish NPP	
		Newborn female	30-year-old male	Newborn female	30-year-old male	Newborn female	30-year-old male
1 y	*CUMLAR*_*av,indoor*_ (%)	0.28	0.019	0.36	0.024	0.33	0.021
	*CUMLAR*_*av,evac*_ (%)	7.28	0.51	8.80	0.65	10.5	0.81
	*CUMLAR*_*res*_ (%)	14.3	2.25	16.5	2.53	18.7	2.82
	*CED*_*res*_ (mSv)	148.6	130.9	173.6	151.8	196.2	171.5
5 y	*CUMLAR*_*av,indoor*_ (%)	0.28	0.019	0.36	0.024	0.33	0.021
	*CUMLAR*_*av,evac*_ (%)	15.3	1.49	18.4	1.85	22.0	2.25
	*CUMLAR*_*res*_ (%)	6.26	1.27	6.93	1.33	7.13	1.37
	*CED*_*res*_ (mSv)	82.3	76.5	90.3	83.0	93.2	86.4
10 y	*CUMLAR*_*av,indoor*_ (%)	0.28	0.019	0.36	0.024	0.33	0.021
	*CUMLAR*_*av,evac*_ (%)	18.1	1.97	21.4	2.37	25.3	2.81
	*CUMLAR*_*res*_ (%)	3.47	0.79	3.92	0.82	3.88	0.81
	*CED*_*res*_ (mSv)	51.9	49.3	56.9	53.2	56.4	53.7

In Table 11, the high impact of age on the averted risk by this protective measure can be seen, where, regardless of the fallout scenario, the *CUMLAR*_*av*_ for a newborn female can be more than a factor 10 higher compared with a male adult.

#### *CED*_*res*_* and CUMLAR*_*av*_* for residents in protective action scenario (3) in *Table 7

*CED*_*res*_ for three NPP fallout scenarios, decontamination with 50% efficiency, for four different ecological half-times for radiocaesium, *T*_*eco,Cs,long*_, assuming wet deposition, is given for various resettlement times in Table 12. The calculations are based on the mitigated accident Scenario (3) in Table 7. It can be seen that *CED*_*res*_ can vary by up to a factor of 2 for short resettlement times between a very short and very long *T*_*eco,Cs,long*_, and that the relative difference increases for longer resettlement times.

**Table 12:** *CED*_*res*_ (mSv) as a function of resettlement time, *t*_*ret*_, and long-term ecological half-time of caesium, *T*_*eco,Cs,long*_, assuming protective action scenario (3) in Table 7 with 50% decontamination after evacuation. Residual dose computed for *c*_*short,i*_ = 0 (conservative assumption for all nuclides), 3 days’ sheltering, and assuming wet deposition.

*t*_*ret*_	NPP Fallout scenario											
	Chernobyl 1				Fukushima				Swedish NPP			
	*T*_*eco,Cs,long*_											
	3.2 y	6.7 y	15 y	60 y	3.2 y	6.7 y	15 y	60 y	3.2 y	6.7 y	15 y	60 y
0 y	61.7	74.1	92.5	120.9	74.8	88.5	108.0	137.1	86.4	101.7	122.3	152.3
1 y	45.5	57.4	75.6	103.9	52.3	65.5	84.7	113.7	58.9	73.6	93.9	123.7
5 y	25.3	34.0	50.1	76.8	26.9	36.0	52.5	79.6	27.7	37.3	54.2	81.7
10 y	17.4	22.6	35.4	59.4	18.3	23.6	36.5	60.5	18.3	23.7	36.6	60.8
15 y	13.1	16.0	25.8	46.4	13.9	16.9	26.6	47.3	13.8	16.8	26.5	47.3

Table 13 gives *CUMLAR*_*av*_ for various relocation times, using protective action scenario (3) in Table 7, The consistently higher averted risk for newborn female compared to adult (average of 30-year-old old male and female) (almost a factor 7) can be clearly seen for short resettlement times, although this difference decreases somewhat with increasing *t*_*res*_. The higher ^134^Cs/^137^Cs ratio for fallout event Fukushima and Swedish NPP results in a substantially higher benefit of evacuation, in terms of averted risk, especially for newborn female.

**Table 13:** *CUMLAR*_*av*_ (%) of total cancer (excluding non-fatal skin cancers) from external ground exposure for newborn female and 30-year-old adult for various resettlement time, using *T*_*eco,Cs,long*_ = 6.7 y, *c*_*short,Cs*_ = 0, with 3 days’ sheltering, and assuming wet deposition.

*t*_*ret*_	NPP Fallout scenario					
	Chernobyl 1		Fukushima		Swedish NPP	
	Newborn female	30-year-old adult	Newborn female	30-year-old adult	Newborn female	30-year-old adult
1 y	1.87	0.24	2.54	0.33	3.14	0.41
2 y	2.88	0.39	3.89	0.53	4.89	0.67
5 y	4.47	0.66	5.90	0.87	7.35	1.08
10 y	5.41	0.85	6.93	1.08	8.50	1.32

#### External dose from groundshine: Initial local deposition density vs. reference level of 100 mSv

LARCalc can be used to calculate which *A*_*dep,Cs-137,loc*_ values are compatible with evacuation when considering various protective measures. This *A*_*dep,Cs-137,loc*_ value can then be used as an initial operational intervention level for e.g. evacuation. Table 14 gives the corresponding initial deposition density values for ^137^Cs that will give rise to an external dose contribution from groundshine exceeding 100 mSv for a certain resettlement time, *t*_*ret*_.

**Table 14:** Initial local deposition density of ^137^Cs (kBq/m^2^), *A*_*dep,Cs-137.loc*_, at which a certain resettlement time will ensure compliance with the reference value *CED*_*res*_ = 100 mSv from external dose, calculated for three fallout scenarios and for four values of *T*_*eco,Cs,long*_.

*t*_*ret*_	NPP Fallout scenario*											
	Chernobyl 1				Fukushima				Swedish NPP			
	*T*_eco,Cs,long_**											
	3.2 y	6.7 y	15 y	60 y	3.2 y	6.7 y	15 y	60 y	3.2 y	6.7 y	15 y	60 y
0 y	2429	1514	972	627	1893	840	1246	564	1559	1056	736	510
1 y	3582	1928	1133	691	2903	1006	1640	635	2429	1417	900	585
2 y	4930	2334	1274	744	4131	1160	2046	697	3578	1819	1062	654
5 y	11,314	3807	1712	894	10,126	1636	3552	867	9671	3383	1576	844
10 y	32,723	7436	2562	1151	29,004	2517	7159	1139	31,420	7223	2512	1135

*Scenario includes a wet deposition with a 3 days´ sheltering period.

**For the *T*_*eco*_-values a conservatively assumption of *c*_*short,i*_ = 0 has been used.

#### *CED*_*res*_* and CUMLAR*_*av*_* for residents in protective action scenario (3) and (4) in *Table 7

*CED*_*res*_ resulting from fictitious fallout from a Swedish NPP, assuming wet deposition, decontamination with 50% efficiency for three different ecological half-times, *T*_*eco,Cs,long*_, are given in Table 15 . The calculations are based on the mitigated accident Scenario (4) in Table 7. From Table 15 it can be seen how the residual dose increases with an increasing *T*_*eco,Cs,long*_. Assuming a similar decontamination efficiency to that in Japan after the Fukushima accident, the residual dose would have been much higher if *T*_*eco,Cs,long*_ had been similar to that found in the Russian rural settings after the Chernobyl accident (around 15 y)^46,60^.

**Table 15:** *CED*_*res*_ (mSv) for decontamination efficiencies, *S*_*decont*_ of 50% and 90%, and for three different values of *T*_*eco,Cs,long*_ (Table 3), with 3 days’ sheltering. NPP fallout scenario: Fictious Swedish NPP fallout scenario (Table 2), assuming wet deposition and extensive food restrictions, *S*_*aliment*_ = 0.

*t*_*ret*_	*T*_*eco,Cs,long*_					
	3.2 y		6.7 y		15 y	
	50%	90%	50%	90%	50%	90%
1 y	58.9	42.8	73.6	45.7	93.9	49.8
5 y	27.6	23.9	37.3	25.8	54.2	29.2
10 y	18.3	17.4	23.6	18.5	36.6	21.1
30 y	6.72	6.71	7.17	6.80	10.48	7.47

**Figure 5:**
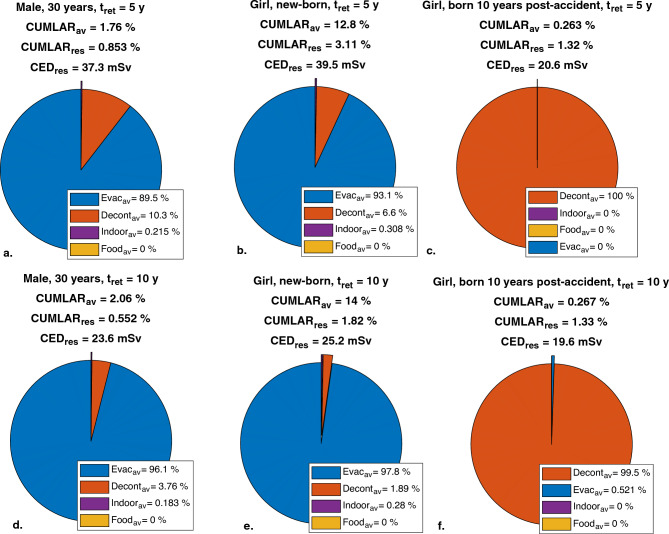
Pie charts showing the *CUMLAR*_*av*_ for two different evacuation times, for an adult male, a newborn female and female born 10 years after the accident. The fallout scenario was Swedish NPP (Table 2), with *T*_*eco,Cs,long*_ = 6.7 y, assuming 3 days’ sheltering, decontamination with 50% efficiency performed after evacuation. Note that *Evac*_*av*_, *Decont*_*av*_, *Indoor*_*av*_ and *Food*_*av*_ are given as percent of the total averted risk, and that averted risk, *CUMLAR*_*av*_, are given as percental points.

The *CUMLAR*_*av*_ for Scenario (3) in Table 7 for the fictious Swedish NPP fallout scenario (Table 2) is illustrated in pie charts for a 30-year-old male, a newborn female, and a female born 10 years after the accident in Fig. 5. In this scenario, a value of *T*_*eco,Cs,long*_ = 6.7 y*,* and a decontamination efficiency of either 50% or 90% were used. It can be seen that in relative terms, evacuation will have a proportionally higher protective effect on newborn female than male adult.

#### *CED*_*res*_* and CUMLAR*_*av*_* for residents in protective action scenario (5) in *Table 7

Values of *CED*_*res*_ for three NPP fallout scenarios (Table 2) and various resettlement times, combined with different durations of food restrictions are given in Table 16. Lifting food restrictions 10 y after the accident can result in up to twice as high a *CED*_*res*_ to the resettled population, compared with when lifted after 30 years. Another observation is that extensive food restrictions (*S*_*aliment*_ = 0) will generally lead to less reduction in the radiation detriment than decontamination for future generations of newborns in the affected area.

**Table 16:** *CED*_*res*_ (mSv) for various food restriction times, and resettlement times for typical urban populations, for three different fallout scenarios (Table 2) assuming extensive food restrictions (*S*_*aliment*_ = 0), 3 days’ sheltering, and assuming wet deposition. Decontamination with 50% efficiency performed after evacuation.

*t*_*food*_	Fallout scenario								
	Chernobyl 1			Fukushima			Swedish NPP		
	*t*_*ret*_								
	1 y	5 y	10 y	1 y	5 y	10 y	1 y	5 y	10 y
10 y	50.3	29.8	21.9	50.3	29.8	21.9	50.3	29.8	21.9
20 y	43.8	23.3	15.4	43.8	23.3	15.4	43.8	23.3	15.4
30 y	39.8	19.3	11.4	39.8	19.3	11.4	39.8	19.3	11.4

**Figure 6:**
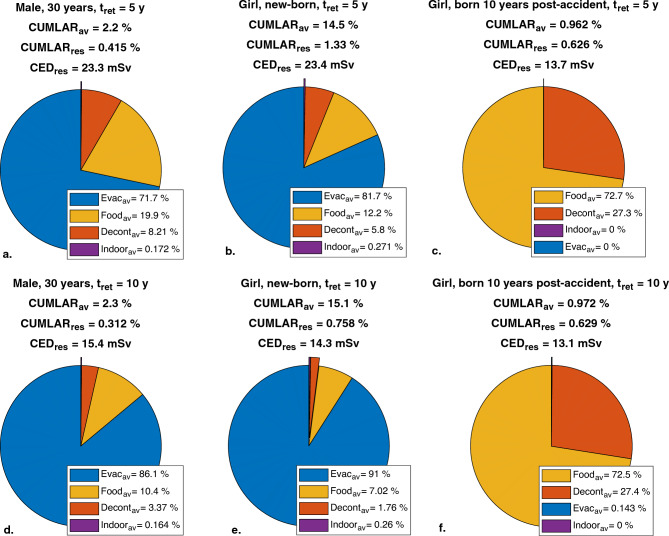
Pie charts showing the averted risk, *CUMLAR*_*av*_, for two different evacuation times, for an adult male, a newborn girl and a female born 10 years after the accident. The fallout scenario was Swedish NPP (Table 2). Results are given for 3 days’ sheltering, 20 years of food restrictions (*S*_*aliment*_ = 0) and decontamination with 50% efficiency performed after evacuation. *CED*_*res*_, *CUMLAR*_*res*_ and *CUMLAR*_*av*_ are included. Note that *Evac*_*av*_, *Decont*_*av*_, *Indoor*_*av*_ and *Food*_*av*_ are presented in terms of their relative contribution to the total averted risk, *CUMLAR*_*av*_, whereas *CUMLAR*_*av*_ and *CUMLAR*_*res*_ are given as percental points of lifetime cancer risk.

The *CUMLAR*_*av*_ from a fictitious Swedish NPP release event in relation to the protective measures implemented (according to Scenario (5) in Table 7) is illustrated as pie charts for a 30-year-old male, a newborn female, and a female born 10 years after the accident in Fig. 6.

### Inclusion of ^131^I in milk, aggregate transfer of ^90^Sr/^90^Y and ^89^Sr, and initial inhalation dose in LARCalc

To illustrate the impact of the added exposure pathways in LARCalc including the dose from plume inhalation, their contributions to the unmitigated *CED* (mSv) are given in Table 17. Here the residual doses, *CED*_*res*_ (mSv), are presented for a 30-year-old male for three different NPP fallout scenarios using protective action scenario (1) in Table 7 when including 1 MBq m^2^ of ^90^Sr/^90^Y ground deposition. Values are presented for dry deposition, with the default values: *f*_*shield*_ = 0.4, *f*_*out*_ = 0.2, *f*_*snow*_ = 1, inhalation absorption type “Medium” and a 24-h plume. The corresponding values for the unmitigated *CUMLAR*_*res*_ are given in Table 18.

**Table 17:** *CED* (mSv) without any protective measures for dry deposition from different NPP fallout scenarios (Table 2) and including 1 MBq m^-2^ ^90^Sr, for a 30-year-old male at the time of the accident and a 1-year-old female.

Exposure pathway	NPP fallout scenarios					
	Chernobyl 1		Fukushima		Swedish NPP	
	1-year-old female	30-year-old male	1-year-old female	30-year-old male	1-year-old female	30-year-old male
Groundshine	140.5	117.9	172.8	140.9	203.0	164.6
Cs transfer	29.4	34.2	32.8	38.5	36.6	43.3
Sr transfer	16.2	36.5	16.2	36.5	16.2	36.5
Iodine in milk	17.6	6.5	25.3	9.2	28.8	10.5
Inhalation dose from conservative plume	27.1	16.0	33.4	18.4	35.3	19.0
Cloudshine from conservative plume	0.64	0.55	1.34	1.14	1.19	1.02
Total	231.5	211.5	281.9	244.7	321.1	275.0

**Table 18:** *CUMLAR*_*res*_ (%) without any protective measures for dry deposition from different fallout scenarios (Table 2) including 1 MBq m^-2^ ^90^Sr, for both an adult male (30 years at accident) and a 1-year-old female.

Exposure pathway	NPP fallout scenarios					
	Chernobyl 1		Fukushima		Swedish NPP	
	1-year-old female	30-year-old male	1-year-old female	30-year-old male	1-year-old female	30-year-old male
Groundshine	14.7	1.62	17.0	1.94	19.8	2.27
Cs transfer	3.66	1.12	4.02	1.22	4.42	1.33
Sr transfer	0.12	0.21	0.12	0.21	0.12	0.21
Iodine in milk	1.36	0.01	1.95	0.02	2.22	0.02
Inhalation dose from conservative plume	1.82	0.10	2.30	0.10	2.52	0.10
Cloudshine from conservative plume	0.06	0.01	0.12	0.01	0.11	0.01
Total	21.8	3.07	25.6	3.50	29.1	3.93

It can be seen in Tables 17 and 18 that groundshine will dominate the radiation risk to all age cohorts, and that the increasing ^134^Cs/^137^Cs ratio between the nuclide vectors (Table 2) is reflected in an absolute increase in *CUMLAR*_*res*_ for a given deposition density of *A*_*dep,Cs-137,reg*_. It can also be seen that the relative contributions from Sr transfer will be more significant for adults than children, as the build-up of Sr is related to the total mass of calcium in the bones^10^. Furthermore, iodine transfer in milk will have a higher impact on *CUMLAR*_*res*_ in children.

### Validation with other models and observations

**Figure 7:**
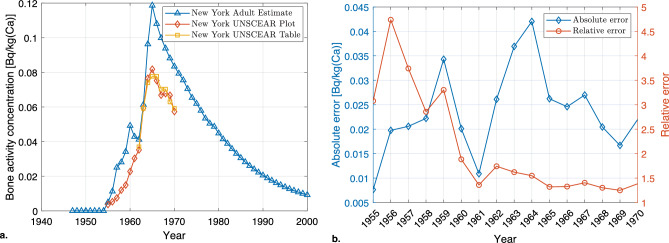
(**a**) Estimate using the strontium model with the IGFD fallout data^61^ as input, averaged over each year, and measured bone activity concentration^62^. (**b**) Absolute and relative error between the modelled estimate and UNSCEAR data^62^.

The strontium transfer described in Eq. (16) was compared with the monthly fallout of ^90^Sr in New York during the years 1950 to 2000 given in The Integrated Global Fallout Database (IGFD)^61^, in order to estimate the activity concentration in bones for adults. As the data in the IGFD are monthly averages, the average activity concentration was calculated for each year, and compared with the data published by UNSCEAR^62^. The results are given in Fig. 7. The average absolute error between the model and the UNSCEAR data is 0.024 Bq/kg(Ca) while the LARCalc average relative error is a factor 2.12 times larger the UNSCEAR data, for the period 1955 to 1970 (seen in Fig. 7b).

A comparison with the European COSYMA model for the transfer of fission products to man, presented by Andersson et al.^63^, shows a factor 2–3 agreement with the present LARCalc model, as can be seen in Table 19.

**Table 19:** Long-term dose contribution to adults (mSv/(MBq m^-2^)) (integrated to age 80 years, average for male and female, 30 years old at accident) assuming wet deposition occurring at the beginning of the growth season. Calculations made with the European COSYMA model reported by Andersson et al.^63^ and LARCalc.

	^137^Cs (mSv/(MBq m^-2^))		^90^Sr (mSv/(MBq m^-2^))	
	COSYMA	LARCalc	COSYMA	LARCalc
Internal effective dose	12.4	23.4	81.0	32.7
External effective dose	8.6	39.5	0	0.040

LARCalc predicted a 4.6 times higher external dose from ^137^Cs than COSYMA. This discrepancy may be due to the use of coefficients from the ICRP^14^ in LARCalc, which were not available at the time of the study by Andersson et al.^63^. Using the coefficients given by the ICRP^14^ to calculate the dose for constant outdoor exposure (i.e. excluding all types of shielding) to ^137^Cs, only taking into account for the physical- and ecological half-time of 6.7 y and assuming wet deposition, gives a *CED* of 84.5 mSv.

Results from LARCalc have been compared with observations made in Russia, in a study by Isaksson et al.^8^. It was found that the Russian data on the aggregate transfer of radiocaesium from the 1990s were between the *T*_*ag,max,Cs*_*(t)* values obtained for hunters and urban residents in Sweden using LARCalc. A more detailed comparison between the *T*_*ag,max,Cs*_ values for radiocaesium in the Nordic countries has been performed by Hjellström et al.^19^.

### Sensitivity and uncertainty analysis

**Figure 8:**
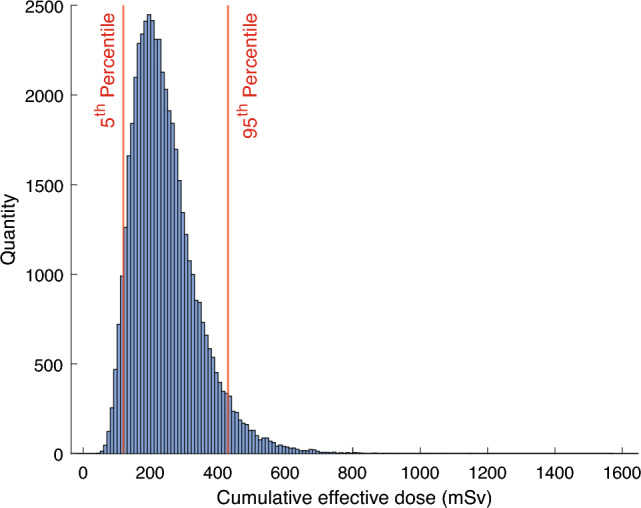
Histogram of the *CED* for an adult male (30 years-old at accident, until age of 80) living in an area affected by fallout containing a nuclide vector according to the Chernobyl 1 (Table 2) without any protective measures. The parameters used in the computations are based on the uncertainties given in Table 20.

An uncertainty analysis was performed in the present study in which each parameter was sampled from a probability distribution (see Table 20). Parameters included in the underlying models but not listed in Table 20 were assumed to have no uncertainty, and thus had fixed values in this analysis. Results for the *CED* to an adult male (aged 30 y at the time of the fallout) and up to the age of 80 y, are presented in Fig. 8, following the Chernobyl fallout in Sweden (Chernobyl 1 in Table 2), normalized to *A*_*dep,Cs-137,reg*_ and *A*_*dep,Cs-137,loc*_ = 1 MBq/m^2 137^Cs. The nuclide vector were edited to *A*_*dep,Sr-90*_ = 1 (resulting in 1 MBq/m^2^ of ^90^Sr) to include the strontium model, thus the doses are comparable to those in Tables 18 and 19. The simulation was performed with 50,000 samples, and yielded a median *CED* of 225.8 mSv with a 5^th^ percentile of 118.8 mSv and a 95^th^ percentile of 429.5 mSv (note that the mean of the doses will be overestimated due to the distributions, as there is a greater probability of a higher value for both lognormal and uniform distributions). The values given in Table 20 were based on uncertainties considered to be appropriate for each parameter (uncertainties briefly described in Isaksson et al.^8^).

**Table 20:** Parameter probability distributions used in uncertainty and sensitivity analysis. Empty cells indicate parameters that are not applicable for the respective type of distribution.

Parameter	Mean or central estimate	SD	Min	Max
**Normal distribution**				
*t*_*1*_	1	0.025		
*t*_*2*_	0.75	0.0188		
*t*_*3*_	15	0.375		
*c*_*1*_	1	0.125		
*c*_*2*_	0.1	0.0125		
*w*	77.5	19,375		
*m*_*Ca,male*_*(adult)*	1.2	0.03		
*a(adult)*	150	3.75		
*INH(adult)*	15.52	0.388		
**Lognormal distribution**				
*A*_*dep,Cs-137,loc*_	6.818	0.42		
*A*_*dep,Cs-137,reg*_	6.818	0.42		
*T*_*eco,i,long*_	LN(*T*_*eco,i,long*_ ⋅0.9141)	0.42		
*T*_*eco,i,short*_	LN(*T*_*eco,i,short*_⋅0.9141)	0.42		
*c*_*short,i*_	LN(*c*_*short,i*_⋅0.9141)	0.42		
*T*_*ag,max,Cs*_	1.812	0.42		
*T*_*eff,Grass*_	1.369	0.42		
**Uniform distribution**				
*A*_*dep,i*_*(t = 0)*			50%	200%
*T*_*ag,Sr*_*(< 29)*			0.1179	0.4716
*T*_*ag,Sr,long*_*(< 29)*			0.0832	0.3328
*T*_*eco,Sr,short*_*(< 29)*			0.14382	0.17578
*T*_*eco,Sr,long*_*(< 29)*			7.79886	9.53194
*k*_*delay*_			0.5009	0.9172
**Triangular distribution**				
*f*_*snow*_				
*f*_*out*_	0.9		0.8	1
*f*_*shield*_	0.2		0.1	0.3
*f*_*shield,inh*_	0.4		0.25	0.55
*TF*_*milk,grass*_	0.5		0.4	0.6
*f*_*inter*_	0.274		0.137	0.548

**Figure 9:**
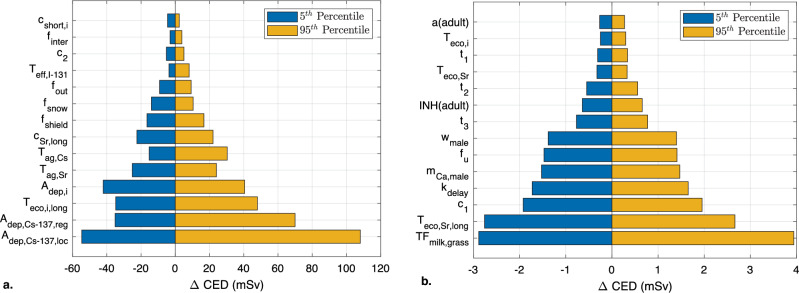
Results of the first sensitivity analysis of the total *CED* for adult male (30 years) up to the age of 80 y. The 5^th^ (blue) and 95^th^ percentiles (yellow) of the 5,000 samples are shown for each parameter. (**a**): results for the 14 most dominant parameters. (**b**): results for the 14 least dominant parameters.

**Figure 10:**
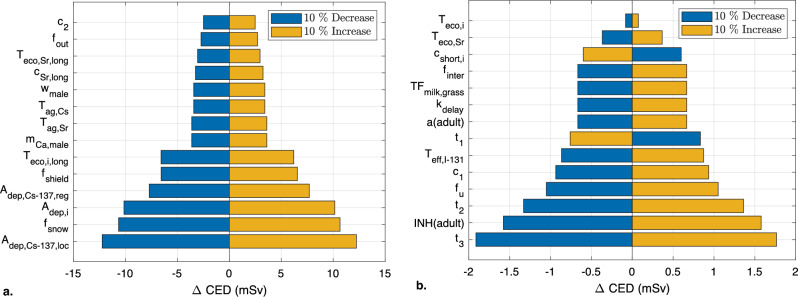
Results of the second sensitivity analysis of the total *CED* for adult male (30 years) up to the age of 80 y. The results for a 10% decrease (blue) and a 10% increase (yellow) in each parameter. (**a**): results for the 14 most dominant parameters. (**b**): results for the 14 least dominant parameters.

Two sensitivity analyses were performed on the LARCalc tool. In the first, each parameter was randomized 5000 times and sampled from the distributions given in Table 20. In the second, each parameter was varied ± 10% from the default value. Both analyses were performed on *CED,* including all pathways, without any protective measures, for an adult male (aged 30 years at the time of the accident) up to the age of 80 years. Figure 9 shows the absolute change in *CED* for 5^th^ and 95^th^ percentiles from the sample distribution of each parameter in the first analysis, while Fig. 10 shows absolute change in *CED* when each parameter was varied. It should be noted that *A*_*dep,i*_ and *c*_*short,i*_ affect all nuclides in the nuclide vector except ^*137*^*Cs*.

The results of the sensitivity analyses show that the parameters that directly affect the fallout, such as *A*_*dep,Cs-137,loc*_, *A*_*dep,Cs-137,reg*_ and *A*_*dep,i*_, and the shielding effect of remaining indoors, *f*_*shield*_, have considerable impact on *CED*. On the other hand, parameters that have a short-term effect, such as *T*_*eco,i,short*_ and *c*_*short,i*_, and the body weight (in this analysis, for a male), *w*_*male*_, have a smaller effect on the *CED*.

## Future work with Larcalc

At the same time as this work was being carried out, the ICRP was updating the dose coefficients for members of the public (ingestion and inhalation of nuclides). Our plan is to include these dose coefficients in an updated version of LARCalc. We also intend to include our own future work on e.g. nuclear weapons fallout and accident releases from the European Spallation Source (ESS) to make LARCalc a more complete tool for estimating doses and risks from different pathways of radiation exposure. There are also plans on making the tool more available as a python program or as an online-based web calculation tool.

## Conclusions

The LARCalc easy-to-use tool is based on models for the prediction of radiation exposures and the associated *LAR* resulting from an atmospheric release of radionuclides from NPP. LARCalc is intended to be used as a training tool for decision makers and can facilitate visualization of how different protective actions can affect the dose and lifetime cancer risk to populations in the event of an NPP emission. In its current version, it is designed to relate the *CED* and *CUMLAR*_*res*_ to the initial local and regional average ground deposition of ^137^Cs. The tool is based on previously published models^8–10^. This paper presents a number of extended features, including the implementation of dose conversion factors presented by the ICRP^14^ to obtain relationships between equivalent doses for various organs and ground deposition of a gamma-emitting radionuclides at various depths, as well as inhalation doses and the contribution to the internal dose from radionuclides such as ^131^I and ^90^Sr/^90^Y.

Furthermore, various doses can now be computed for a wider range of NPP fallout scenarios and their associated nuclide vectors. The user can customize the event after a specific fallout scenario by editing the nuclide vector and ^137^Cs deposition.

This paper has also provided examples of ways in which LARCalc can be used to estimate the projected cumulative effective dose (*CED*), the residual dose (*CED*_*res*_), and the cumulative averted lifetime attributable risk (*CUMLAR*_*av*_) for various combinations of protective measures. The target audience for LARCalc comprises decision-makers involved in emergency preparedness planning at authorities.

The LARCalc tool has been developed for Swedish conditions. For use in other countries, it is recommended that the parameter *T*_*ag,max,Cs*_ and its related time parameters (presented in Table 1) be adapted to better match locally or regionally recorded body burdens of radiocaesium. The sheltering factor and occupancy factor may also need to be adjusted in other countries.

## Data Availability

All data generated or analysed during this study are included in this published article.
